# Grafted Neural Precursors Integrate Into Mouse Striatum, Differentiate and Promote Recovery of Function Through Release of Erythropoietin in MPTP-Treated Mice

**DOI:** 10.1177/1759091416676147

**Published:** 2016-10-27

**Authors:** Stephana Carelli, Toniella Giallongo, Cristina Viaggi, Zuzana Gombalova, Elisa Latorre, Massimiliano Mazza, Francesca Vaglini, Anna Maria Di Giulio, Alfredo Gorio

**Affiliations:** 1 Laboratories of Pharmacology, Department of Health Sciences, University of Milan, Italy; 2 Dipartimento di Ricerca Traslazionale e delle Nuove Tecnologie in Medicina e Chirurgia, Università di Pisa, Italia; 3 Experimental Oncology Department, European Institute of Oncology, Milan, Italy

**Keywords:** adult neural stem cells, cell therapy, erythropoietin, MPTP, Parkinson’s disease, regenerative medicine

## Abstract

Erythropoietin-releasing neural precursor cells (Er-NPCs) are a subclass of subventricular zone-derived neural progenitors, capable of surviving for 6 hr after death of donor. They present higher neural differentiation. Here, Er-NPCs were studied in animal model of Parkinson’s disease. Dopaminergic degeneration was caused by 1-methyl-4-phenyl-1,2,3,6-tetrahydropyridine intraperitoneal administration in C57BL/6 mice. The loss of function was evaluated by specific behavioral tests. Er-NPCs (2.5 × 10^5^) expressing the green fluorescent protein were administered by stereotaxic injection unilaterally in the left striatum. At the end of observational research period (2 weeks), most of the transplanted Er-NPCs were located in the striatum, while several had migrated ventrally and caudally from the injection site, up to ipsilateral and contralateral substantia nigra. Most of transplanted cells had differentiated into dopaminergic, cholinergic, or GABAergic neurons. Er-NPCs administration also promoted a rapid functional improvement that was already evident at the third day after cells administration. This was accompanied by enhanced survival of nigral neurons. These effects were likely promoted by Er-NPCs-released erythropoietin (EPO), since the injection of Er-NPCs in association with anti-EPO or anti-EPOR antibodies had completely neutralized the recovery of function. In addition, intrastriatal administration of recombinant EPO mimics the effects of Er-NPCs. We suggest that Er-NPCs, and cells with similar properties, may represent good candidates for cellular therapy in neurodegenerative disorders of this kind.

## Introduction

Restorative therapies based on neural cell replacement have been extensively studied, and in particular, it is considered of interest in conditions such as the loss of substantia nigra (SN) dopaminergic neurons in Parkinson’s disease (PD; Barker et al., 2013; Barker, 2014). Transplantation of dopamine-containing neurons (Bjorklund et al., 2002; Kim et al., 2002; Yang et al., 2008; Kriks et al., 2011) and allografts of ventral mesencephalon tissue received and made synapses within the host brain and promoted recovery affection in animal models of PD (Schultzberg et al., 1984; Lindvall at al., 1990; Haque et al., 1997; Takahashi et al., 2007; Martìnez-Cerdeño et al., 2010). In pioneer human clinical trials, dopaminergic neurons transplanted into the caudate putamen promoted the reinnervation of striatum, with restored dopamine (DA) release. But the clinical outcome had been highly variable (Barker et al., 2013; Barker, 2014), and unfortunately a significant number of these patients had developed quite debilitating side effects, such as dyskinesias of orofacial muscles and upper and lower extremities (Barker, 2014; Hallett et al., 2014; Kefalopoulou et al., 2014; Hallett et al., 2015). In addition, as predicted, the use of human brain specimens gave rise to numerous ethical concerns (Barker and de Beaufort, 2013; Barker, 2014), and the combined result of ethical issues and severe side effects forced new efforts toward other cellular sources, such as dopaminergic neurons, obtained from induced pluripotent stem cells through somatic cell reprogramming (Yu et al., 2007; Wernig et al., 2008; Sundberg et al., 2013; Doi et al., 2014).

Recently, we reported the existence of a subclass of subventricular zone-derived neural progenitors (erythropoietin-releasing neural precursors cells [Er-NPCs]) surviving after donor death, with differentiation dependent upon autocrine release of erythropoietin (EPO; Marfia et al., 2011). When Er-NPCs, formerly called PM-NPCs, were applied intravenous after traumatic spinal cord injury in the mouse, the treatment resulted in a marked attenuation of secondary degeneration and neuroinflammation associated to a significant improvement of hind limb function (Carelli et al., 2014, 2015). Most of transplanted Er-NPCs had become cholinergic and showed great morphological differentiation (Carelli et al., 2015). Here, we explored the therapeutic potential of Er-NPCs in a preclinical model of PD. Er-NPCs were infused unilaterally into striatum of symptomatic and dopamine-depleted 1-methyl-4-phenyl-1,2,3,6-tetrahydropyridine (MPTP)-exposed C57BL/6 mice. We observed that more than 75% of injected Er-NPCs were vital and capable of migrating ventrally and caudally throughout the striatum, and ipsilateral and contralateral SN pars compacta (SNpc) were also reached. Transplanted Er-NPCs differentiated morphologically and biochemically being positive to microtubule-associated protein 2 (MAP-2), neuronal nuclei (NeuN), and tyrosine hydroxylase (TH). All these features and effects are likely dependent on its own-released EPO, since all of these were abolished by the coinjection of Er-NPCs with anti-EPO or anti-EPO-R antibodies. Finally, we show that Er-NPCs-treated animals improved their typical Parkinsonism within the 3 days after cell transplantation, and this was accompanied by significant sparing of SN neurons.

## Materials and Methods

### Animals and Study Approval

For this study, we used adult C57BL/6 male mice (Charles River, Milan, Italy), 3 to 4 months old and weighing 20 to 24 g. The animals were kept for at least 7 days before the experiments were carried out in standard conditions (22 ± 2℃, 65% humidity, and artificial light between 08:00 a.m. and 08:00 p.m.) with food and water *ad libitum.* Moreover, mice were trained for 1 week before the MPTP treatment, in order to educate them to behavioral testing.

All of the procedures were performed by following the Italian Guidelines for Laboratory Animals, which conform to the European Communities Directive of September 2010 (2010/63/UE), and the Review Committee of the University of Milan approved the work.

### Er-NPCs Isolation

Er-NPCs constitutively expressing green fluorescent protein (GFP) were obtained from adult C57BL/6-Tg(UBC-GFP)30Scha/J mice weighing 25 to 30 g (Charles River) as described earlier (Marfia et al., 2011; Carelli et al., 2014, 2015).

### Animal Treatments

**Figure 1.**
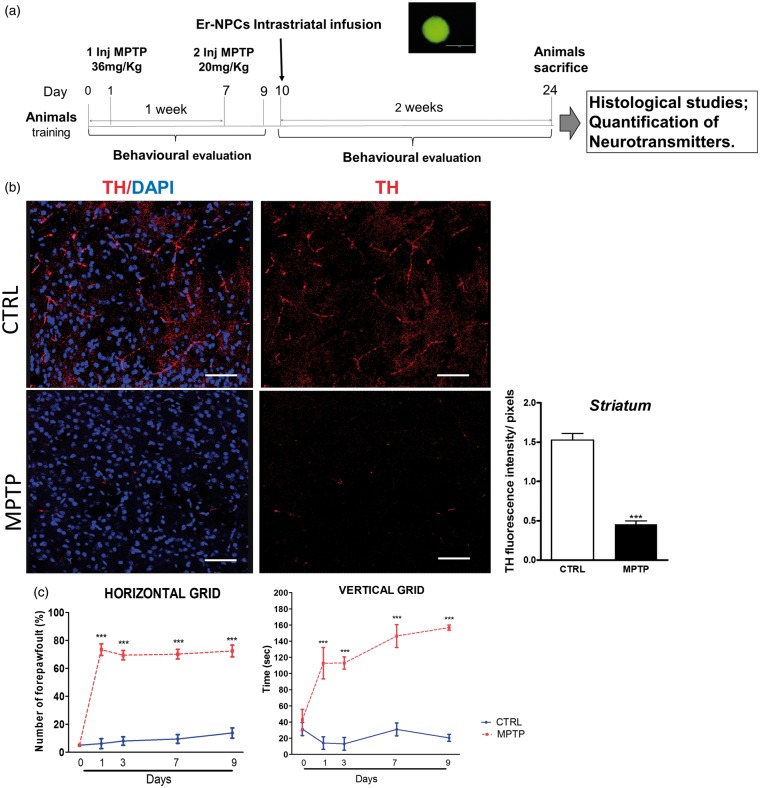
MPTP paradigm applied reduces nigrostriatal dopamine neurons and causes functional disability in mice. (a) Experimental plan set up to investigate the effects of Er-NPCs transplanted in MPTP mouse model of Parkinson’s disease. The photo insert shows an example of neurosphere formed by GFP-expressing-Er-NPCs. (b) Striatal coronal slices of healthy mice (CTRL) and MPTP mice (Day 10 after the first injection of MPTP) stained with anti-TH antibody (Bars = 50 µm). Quantification was made by ImageJ picture analysis software. ***p < .001 versus CTRL. (c) Evaluation of functional impairment was performed by means of two different behavioral tests (inverted and vertical grid, see Materials and Methods section for details). Data are expressed as mean ± *SD* of two independent experiments.

Parkinsonism was induced by the intraperitoneal (IP) administration of MPTP following the acute paradigm with a small modification (Del Zompo et al., 1990; Zuddas et al., 1990; Petroske et al., 2001). Briefly, mice were administered of a double dose of MPTP hydrochloride: a first IP injection of MPTP (36 mg/kg), and after 7 days, the animals were subjected to a second IP injection of MPTP (20 mg/kg). To investigate the stability of the lesion, a group of animals (CTRL, *n* = 8; MPTP, *n* = 8) were sacrificed 3 days after the second toxin administration (Day 10; Figure 1(a)), five animals for each condition were used for DA and metabolites determination (see later), and three animals for each condition were used for histology studies. After 3 days from the second MPTP injection, a group of animals were transplanted with 5 × 10^4^ cells/µl (5 µl) GFP expressing Er-NPCs (*n* = 31), according to the following stereotaxic coordinates in relation to bregma: 0.1 mm posterior, 2.4 mm mediolateral, and 3.6 mm dorsal at the level of left striatum (Cui et al., 2010). Two weeks after transplantation (Day 24; Figure 1(a)), 13 animals from each group (CTRL, MPTP, MPTP + Er-NPCs) were sacrificed by cervical dislocation, and their brains were removed and dissected (see Figure 1(a) for experimental plan). Immediately after dissection, the whole striatum, frontal cortex, and midbrain were frozen on dry ice for neurotransmitters determination (see as follows). Twelve animals for each of the groups CTRL, MPTP, and MPTP + Er-NPCs and nine animals for the group MPTP + Er-NPCs + anti-EPO were anesthetized by an IP injection of sodium pentobarbital (65 mg/kg), perfused through the left ventricle with 50 mL of saline solution, and fixed with 200 mL of 4% paraformaldehyde in 0.1 mol/L phosphate-buffered saline (PBS). Brains were subsequently removed from the skulls and then cryoprotected at 4℃ in sucrose 300 g/L in 0.1 mol/L PBS solution for further sectioning.

### Behavioral Tests

The recovery of motor dysfunction, before and after cell transplantation, was investigated by means of horizontal grid test (Tillerson and Miller, 2003) and vertical grid test (Kim et al., 2010).

#### Horizontal grid test

The grid apparatus was constructed according to Tillerson and Miller (2003). The animal was videotaped for 30 sec, and the videos were replayed for percentage forepaw fault analysis using a recorder with slow motion option. The number of unsuccessful forepaw steps divided by the total number of attempted forepaw steps was evaluated. Before MPTP administration, mice were acclimated to the grid twice a day for 1 week.

#### Vertical grid test

The vertical grid apparatus was constructed according to Kim et al. (2010). For this test, the mouse was placed 3 cm from the top of the apparatus, facing upward, and was videotaped while it turned around and climbed down. The score reported was the time required by the mouse to make a turn, climb down, and reach the bottom of the grid with its forepaw within 180 sec. Before MPTP administration, mice were acclimated to the grid twice a day for 1 week.

### Determination of DA and Metabolites

For the analysis of DA and its metabolites, the protocol described by Vaglini et al. (2004) was used. Briefly, the striatal tissue samples were homogenized in 600 µL ice-cold 0.1 N perchloric acid containing 10 pg/lL dihydroxybenzylamine (DBA) as the internal standard; an aliquot of homogenate was assayed for protein. The homogenates were centrifuged, and the levels of monoamines and their metabolites in the supernatant were determined by reverse-phase high-performance liquid chromatography coupled to an electrochemical detector. One liter of mobile phase contained 10.35 g (75 mM) sodium dihydrogen orthophosphate, 0.505 g (2.5 mM) heptan sulfonic acid, 25 mM EDTA, 100 µL triethylamine, and 200 mL acetonitrile adjusted to a final pH of 3.00 with phosphoric acid. A C18 inertsil ODS-3, 4.6 250 mm, 5 µm, reverse-phase column was used (Beckman, San Ramon, CA, USA). The mobile phase (filtered and degassed) was delivered at a flow rate of 1.2 mL/min; the applied potential was set to −0.10 V (Detector 1) and + 0.30 V (Detector 2). For catecholamine assays, a standard curve was prepared using known amounts of DA and metabolites dissolved in 0.1 N perchloric acid containing a constant amount (10 pg/mL) of the internal standard (DBA) used for tissue samples. The standard curve for each compound (DA or its metabolite) was calculated using regression analysis of the ratios of the peak areas (compound area or DBA area) for various concentrations of each compound recorded at the reducing electrode. An analogous regression analysis was performed for the oxidizing electrode (Vaglini et al., 2004, 2009).

### Immunohistochemistry and Quantitative Analysis

Coronal sections (20 µm) of the whole brain were cut at −25℃ using a cryostat (Leica), and slides were collected onto glass slides and rinsed with PBS, treated with blocking solution (10% NGS, 0.2% Triton X-100) by following our previously published protocols (Carelli et al., 2014, 2015). The following primary antibodies were used: MAP-2 (1:300; Chemicon), NeuN (1:100; Millipore), TH (1:500; Millipore), choline acetyltransferase (ChAT, 1:1000; Chemicon), gamma aminobutyric acid (GABA, 1:500; Millipore), NG2 Chondroitin sulfate proteoglycan (1:200; Millipore), nestin (1:200; Millipore), EPO (1:200; Santa Cruz), monocytes/macrophages (MOMA,1:25; Millipore). The following secondary antibodies were used: 546 goat anti-rabbit IgG (1:200; Alexa), 546 goat anti-mouse IgG (1:200; Alexa), and 546 donkey anti-goat IgG (1:200, Alexa). Images were acquired using standardized confocal microscopy (Leica SP2 confocal microscope with He or Kr and Ar lasers; Heidelberg, Germany). The quantification of GFP-positive transplanted Er-NPCs expressing typical neural markers mentioned earlier was determined by confocal microscopy with the overlap application. The quantification was expressed as percentage of positive cells versus the total number of GFP-positive Er-NPCs. Images of SN immunostaining were acquired in the region corresponding to bregma 2.80/3.52 mm as indicated in the Paxinos and Franklin’s (2001) atlas. Microphotographic digital analysis were performed by using ImageJ software (Jensen, 2013), and without considering the ventral tegmental area (Liang et al., 2004). The quantification of the positive pixels versus the negative background elicits an index score that includes the contributions from fibers and neuronal somata. The immunostaining conditions were the same for all sections within a single brain, in that the staining solutions were the same, as were the reaction times in the solutions. The microscope light intensity of the laser was the same for analyzing all given brain sections and for determining the background optical density.

### RNA Extraction and Real Time-Polymerase Chain Reaction

IL-6 mRNA analyses were performed at 7 days after transplant (*n* = 3 mice for each group) in striatum homogenates as described in Carelli et al., 2015. Briefly, left and right striatum regions were dissected out rapidly, frozen on dry ice, and stored at −80℃ for further analyses. Total RNA was extracted using TRIZOL® reagent (Life Technologies) following manufacturer’s instructions, quantitated, and processed. Primers were designed using Oligo Perfect Designer Software (Life Technologies). AmpliTaq activation 95℃ for 10 min, polymerase chain reaction (PCR) denaturation step 95℃ for 15 sec, PCR annealing step 60℃ for 30 sec, PCR elongation 75℃ for 30 sec, and 40 cycles of PCR were performed. PCR results were normalized versus 18S. Primer sequences were as follows:

**Table:** 

18S	**F:** TTTCGGAACTGAGGCCATGATTAAG
	**R:** AGTTTCAGCTTTGCAACCATACTCC
IL6	**F:** TCCAGTTGCCTTCTTGGGACTGATGCTGGT
	**R:** AGTTTCAGATTGTTTTCTGCAAGTGCATCA

### Statistical Analysis

Statistical analyses between groups were evaluated using GraphPad Prism 4.00 version, and data are expressed as mean ± *SD*. Behavioral data were analyzed with a one-way analysis of variance (ANOVA) model with time and group (CTRL, MPTP, MPTP + Er-NPCs, MPTP + Er-NPCs + anti-EPO, and MPTP + Er-NPCs + anti-EPOR) as factors. The effects of MPTP and the various combined treatments on striatal catecholamine levels were evaluated statistically by using ANOVA with Scheffe-F analysis. The null hypothesis was rejected when *p* < .05. Markers expression data were analyzed in triplicate, and results were expressed as the average of three animals. The expression each marker was analyzed by one-way ANOVA followed by Bonferroni’s multiple comparisons test to assess statistical significance.

## Results

### Experimental Model

The experimental setup of this study is represented in panel A of Figure 1. The effect of MPTP administration on TH-positive neurons and axon terminals into the striatum was evaluated 10 days after the first MPTP administration. Panel B of Figure 1 shows representative TH immunoreactivity in striatum of controls (healthy; CTRL) and MPTP-treated mice. As it is well known, MPTP causes a massive loss of TH-positive cell bodies and axons that was quantified to be over 70% (Figure 1(b)). In support of the histological data, we performed also two behavioral tests, designed for the investigation of the typical functional impairment occurring in Parkinsonian animals (Figure 1(c)). The horizontal grid test allowed the assessment of the forepaw faults in healthy and MPTP-treated mice. The behavioral impairment was already marked as early as 1 day after MPTP administration with 73% of forepaw faults (*p* < .001 vs. CTRL). Through the vertical grid test, we assessed the time required by the mouse to turn around and descend the grid once positioned on the top. Following MPTP administration, mice hesitated and remained on the top of the grid much longer, therefore the time required to turn and descend from the top of the grid was markedly longer than control. The behavioral impairment remained significantly high throughout the experimental period (Figure 1(c)). In addition to the neuropathological changes, we observed that MPTP caused the reduction of striatal DA and related 3,4-dihydroxyphenylacetic acid and homovanillic acid metabolites as expected (Corsini et al., 1985; Table 1).

**Table 1.** Striatal Determination of Dopamine and Metabolites at Day 10 After MPTP Administration.

Neurotransmitters	CTRL (ng/mg of proteins)	MPTP (ng/mg of proteins)
DA	98.61 ± 10.70	47.58 ± 16.20 *p* < .001
DOPAC	9.07 ± 1.20	5.20 ± 2.36 *p* < .001
HVA	14.65 ± 1.83	6.86 ± 2.13 *p* < .001
3-MT	6.85 ± 1.28	6.71 ± 0.56

*Note.* DA = dopamine; DOPAC = 3,4-dihydroxyphenylacetic acid; HVA = homovanillic acid; 3-MT = 3-methoxytyramine; CTRL = control; MPTP = 1-methyl-4-phenyl-1,2,3,6-tetrahydropyridine. Levels of DA, DOPAC, HVA, and 3-MT were quantified in the striatum by using HPLC analysis (see Materials and Methods section). Data are expressed as mean ± *SD* (*n* = 5 mice in each group).

### Localization of Transplanted Er-NPCs

**Figure 2.**
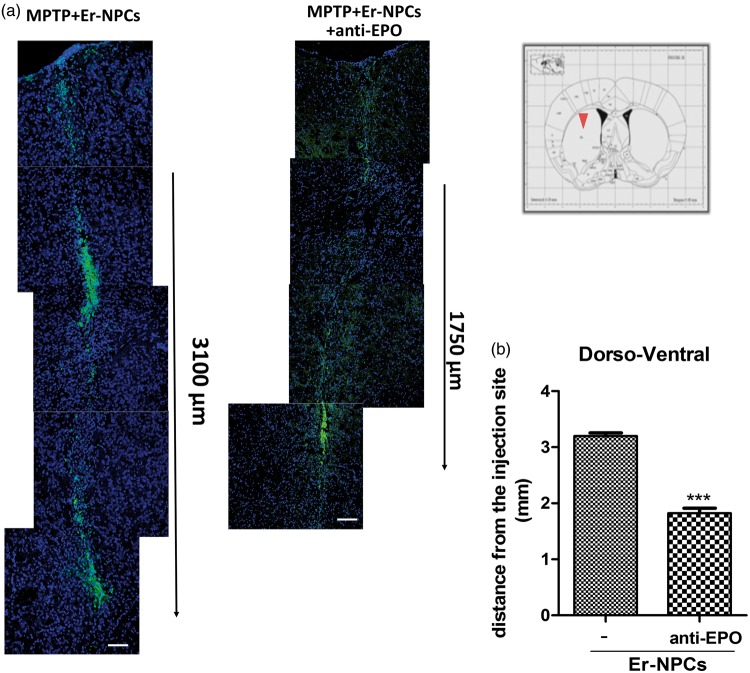
Grafted Er-NPCs migrate throughout the striatum following a dorso-ventral pathway. (a) Confocal images of Er-NPCs intrastrial distribution (green) 2 weeks after transplantation in coronal sections taken from Er-NPCs-injected MPTP mouse brains with or without anti-EPO antibody (Bars = 100 µm). (b) Length of migration from the injection site of Er-NPCs in absence or presence of anti-Epo antibody (distance expressed in mm). Images have been evaluated by confocal picture analysis software. ***p < .001 versus Er-NPCs-transplanted mice in absence of the anti-EPO coinjection. Data refer to one brain for each experimental condition. Comparable results have been observed on other two investigated animals for each experimental condition.

**Figure 3.**
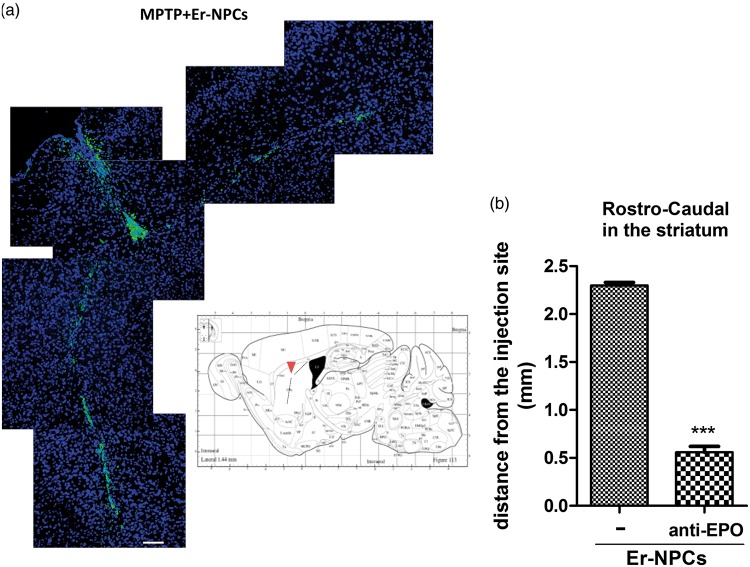
Injected Er-NPCs migrate rostro-caudally throughout the striatum. (a) Sagittal sections of Er-NPCs-injected MPTP mouse brain. Transplanted Er-NPCs (green) migrate for several microns into the striatum following rostro-caudal directions (Bars = 100 µm). The same results have been observed on other two investigated animals with similar approach. (b) Quantification of rostro-caudal length pathway of Er-NPCs injected in absence or presence of anti-EPO antibody. ***p < .001 versus Er-NPCs-transplanted mice. Data refer to one brain for each experimental condition. Comparable results have been observed on other two investigated animals for each experimental condition.

**Figure 4.**
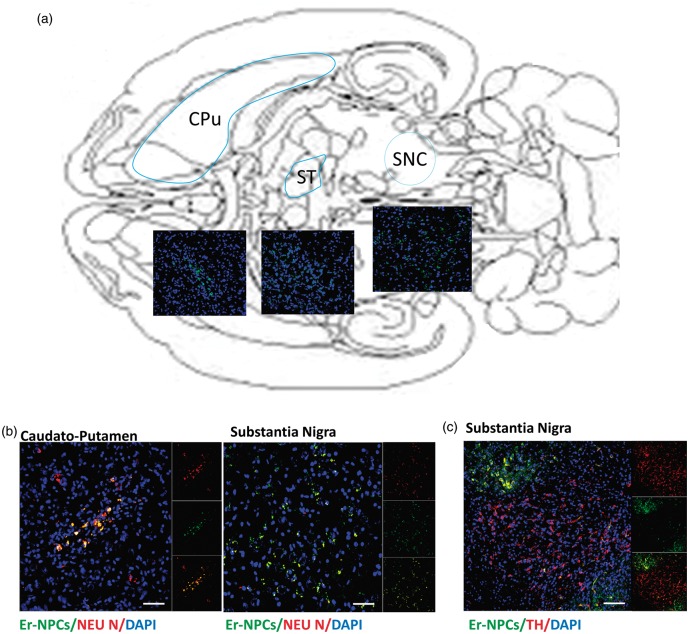
Injected Er-NPCs reach the substantia nigra. (a) Schematic representation of mouse brain transversal section with images representing the distribution of Er-NPCs (green) 2 weeks after transplantation (Day 24 in Figure 1(a)). Er-NPCs migrate rostro-caudally for more than 3 mm reaching the substantia nigra. (b) and (c) Magnification of brain coronal sections obtained from Er-NPCs-injected MPTP mice immunostained with anti-NeuN or anti-TH (red), GFP is showed in green, and nuclei were stained with DAPI (blue; Bars = 100 µm for TH; 50 µm for NeuN). Images are representative of (*n* = 3) investigated brains. CuPu = caudate putamen; ST = subthalamus; SNpc = substantia nigra pars compacta.

Healthy and lesioned animal brains with or without Er-NPCs injection were sectioned. A large portion (78.50 ± 7.51%) of the 2.5 × 10^5^ cells injected into the left striatum of each animal was still present in the brain within 2 weeks after transplantation. The vast majority (69.56 ± 4.5%) of GFP-positive transplanted Er-NPCs were within the striatum (Figure 2(a)). Grafted Er-NPCs appeared rather differentiated with an oval soma and abundant neuritic processes. Previous reports had shown that transplanted stem cells could migrate into the host striatum (Kim et al., 2002; Martinez-Cerdeño et al., 2010); here, we observed that grafted Er-NPCs migrate from the injection site across the striatum (Figure 2(a)). In 2 weeks, Er-NPCs had migrated up to 3100 µm from the site of injection following a ventral direction (Figure 2(a)). In our previous work, we showed that Er-NPCs express and release EPO that exerts an autocrine action regulating the precursor’s differentiation (Marfia et al., 2011). With the aim at investigating the role of EPO in the precursor’s reparative effect, we performed the coinjection of anti-EPO or anti-EPO receptor antibodies at the concentration of 3 µg/ml (Marfia et al., 2011). The coinjection of anti-EPO antibody strongly reduced the dorso-ventral migration of Er-NPCs (Figure 2(a)). The graph represented in Figure 2(b) reports the comparison of the migratory distance covered by Er-NPCs in absence or presence of anti-EPO antibody (Figure 2(b)). The inhibition of EPO significantly reduced the migratory ability of Er-NPCs. The reassembly of serial pictures, obtained from longitudinal sections, by confocal acquisitions (Figure 3(a)) shows that Er-NPCs migrate linearly within the striatum (Figures 2 and 3) also following a rostro-caudal pathway. The coinjection with anti-EPO antibody abolished the migration following such a route suggesting that it might be somewhat regulated by EPO (Figure 3(b)) Through transversal brain sections, we observed that the Er-NPCs migrated beyond the borders of the striatum, and that SN pars compacta, ipsilateral, and contralateral to the injection was reached (Figure 4(a)). Here, Er-NPCs integrate in the SNpc among endogenous TH-positive neurons (Figure 4(b) and (c)). By using references available in adult mouse brain atlas (Paxinos and Franklin 2001; Allen Mouse Brain Atlas available online), we calculated that Er-NPCs travelled for 3280–3360 µm following the rostro-caudal direction. The Er-NPCs localization seemed to be restricted to the brain areas affected by MPTP, we did not find GFP positive Er-NPCs in other nearby regions. Very few cells were found in the cortex probably due to spilling while lowering the needle into the striatum.

### Expression of Typical Markers by Transplanted Er-NPCs

**Figure 5.**
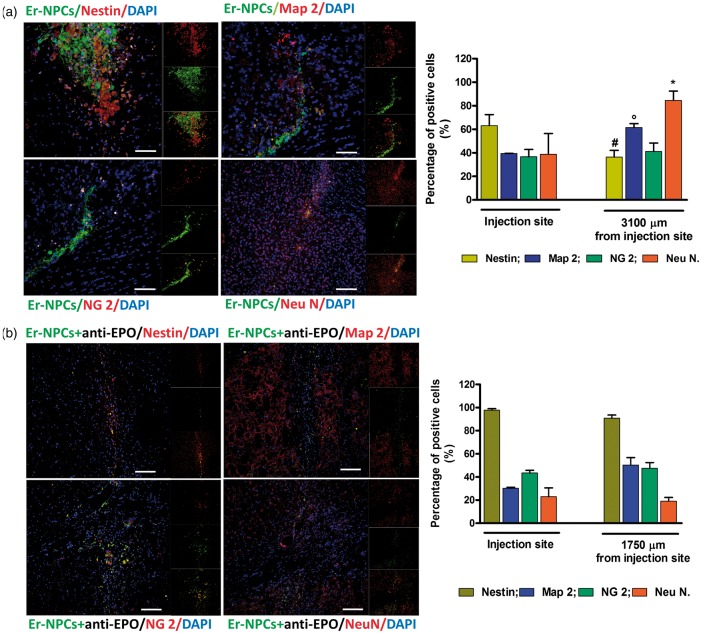
Most Er-NPCs adopted a neuronal fate when transplanted into striatum of MPTP mice. (a) Striatal coronal sections relative to Er-NPCs-transplanted MPTP mice. (b) Striatal coronal sections of Er-NPCs-transplanted MPTP mice in presence of the coinjection of anti-EPO antibody. Sections were immunostained with neural differentiation specific markers (MAP-2, NeuN, NG2, and nestin). The immunostaining is showed in red. Er-NPCs are showed in green (GFP). Nuclei are stained in blue (DAPI). Scale bars represent 50 µm for Nestin, Map-2, and NG2 in panel A. For all other images, bars represent 100 µm. Analysis was performed 2 weeks after Er-NPCs injection. Graphs report the percentage of GFP-positive cells expressing investigated markers in nine different fields for each condition (*n* = 3 mice each group; 3 fields or mouse). Data are expressed as mean ± *SD*. *p < .05 versus NeuN injection site.

Close to the injection site, a higher number of Er-NPCs were positive to nestin (precursor cell marker), while most of the migrated Er-NPCs resulted positively marked by anti-NeuN and anti-MAP-2 antibodies (markers of neuronal differentiation; Figure 5(a)). Distal to the injection site, only a lower percentage of cells was positive to NG2 (marker of oligodendrocytes precursors) and nestin. The number of GFP-positive transplanted Er-NPCs expressing typical neural markers was determined by confocal microscopy. The quantification (expressed as percentage of positive cells) shows that Er-NPCs, migrated from the site of injection, were 84.50 ± 7.97% positive for NeuN and 61.42 ± 8.28% for MAP-2, while 41.13 ± 7.24% were positive for NG2 and 36.41 ± 8.60% for Nestin (Figure 5(a)). Therefore, the EPO-dependent Er-NPCs migration correlates with a higher differentiation as the blockade of EPO action by means of anti-EPO impaired also the *in vivo* differentiation toward neuronal features (Figure 5(b) and Table 2).

**Table 2.** Neural Specific Markers Expression by Er-NPCs Migrating Throughout the Striatum of MPTP Mice.

	Injection site		Migration	
	Er-NPCs (%)	Er-NPCs + anti-EPO (%)	Er-NPCs (%)	Er-NPCs + anti-EPO (%)
Nestin	63.16 ± 9.30	97.79 ± 1.27 (*p* < .001 vs. Er-NPCs)	36.41 ± 5.60	90.86 ± 2.70 (*p* < .001 vs. Er-NPCs)
MAP-2	39.33 ± 0.17	30.28 ± 0.79	61.42 ± 3.28	50.31 ± 6.42
NG2	36.69 ± 6.19	43.41 ± 2.32	41.13 ± 7.24	47.59 ± 4.85
NeuN	38.88 ± 17.57	22.91 ± 7.65	84.50 ± 7.97	19.17 ± 3.06 (*p* < .001 vs. Er-NPCs)

*Note.* MAP-2 = microtubule-associated protein 2; NeuN = neuronal nuclei; Er-NPCs = erythropoietin-releasing neural precursors; EPO = erythropoietin. The comparison refers to Er-NPCs injected alone or in presence of anti-EPO antibody. Quantification of positive cells was made by ImageJ picture analysis software. Data are expressed as mean of three different fields for each condition ± *SD*.

**Figure 6.**
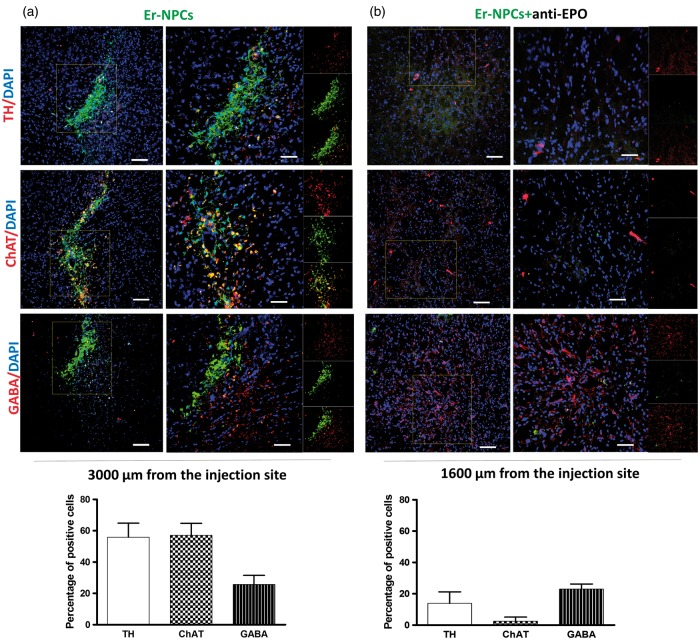
Most Er-NPCs express neuronal lineage markers after transplantation into the striatum of MPTP mice. Confocal images of coronal striatum sections of MPTP mice treated with Er-NPCs (green; GFP) (a) and Er-NPCs + anti-EPO antibody (b). Slices were immunodecorated with anti-TH (TH; red), anti- Choline Acetyltransferase (ChAT; Red), and anti-gamma-aminobutyric acid (GABA; red). Nuclei are stained in blue (DAPI). Scale bars represent 100 µm for the first lane and 50 µm for the second lane. Graphs report the percentage of GFP-positive cells expressing investigated markers in nine different fields for each condition (*n* = 3 mice each group; 3 fields or mouse). Data are expressed as mean ± *SD*.

We also found that a large amount of transplanted cells located distally to the injection site, expressed a marker related to DA synthesis, such as TH (55.90 ± 8.91% in 511 analyzed cells). The cholinergic marker ChAT, which is expressed by striatal excitatory interneurons, was found in 57.03 ± 7.68% of the 489 analyzed cells, while a relative lower amount of cells expressed GABA (25.7 ± 5.38% in 354 analyzed cells; Figure 6). Anti-EPO antibody strongly blocked also the expression of specific neurotransmitter markers, less than 20% of Er-NPCs resulted positively stained for TH, and the expression of ChAT was decreased significantly to less than 3% (Figure 6 and Table 3).

**Table 3.** Percentage of Er-NPCs Positive to TH, ChAT, and GABA.

Markers	Er-NPCs (%)	Er-NPCs + anti-EPO (%)
TH	55.90 ± 8.91	13.80 ± 7.35 *p* < .001
ChAT	57.00 ± 7.68	2.52 ± 2.56 *p* < .001
GABA	25.70 ± 5.38	22.90 ± 3.22

*Note.* TH = tyrosine hydroxylase; ChAT = choline acetyltransferase; GABA = gamma aminobutyric acid; Er-NPCs = erythropoietin-releasing neural precursors; EPO = erythropoietin. The comparison refers to Er-NPCs injected alone or in presence of anti-EPO antibody. Quantification of positive cells was made by ImageJ picture analysis software. Data are expressed as mean of three different fields for each condition ± *SD*.

### Grafted Neural Precursors Express EPO and Regulate EPO Expression in the Recipient Striatum

**Figure 7.**
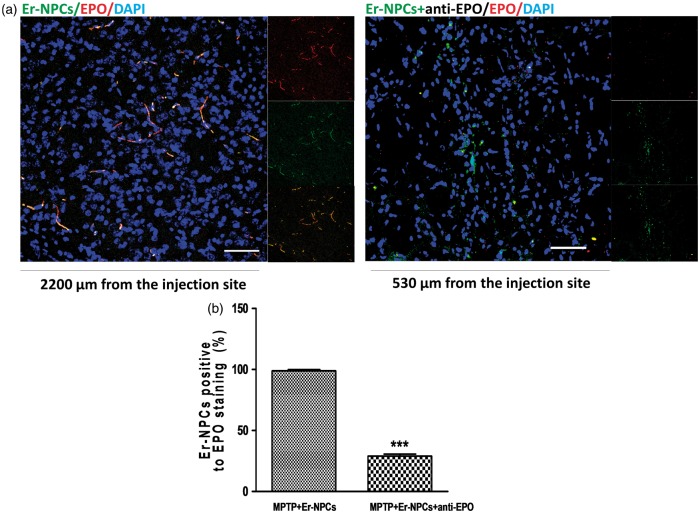
Grafted Er-NPCs express EPO after transplantation. (a) Expression of EPO was investigated by immunofluorescence in striatal coronal sections MPTP animals injected with Er-NPCs in absence or presence of anti-EPO antibody. Analysis was performed 2 weeks after Er-NPCs injection. Er-NPCs are showed in green (GFP), and EPO staining is showed in red, (Bars = 50 µm). (b) The percentage of GFP-cells expressing EPO is reported in the graph. Data are expressed as mean of the quantification performed in three different fields for each condition ± *SD*. ***p < .001.

**Figure 8.**
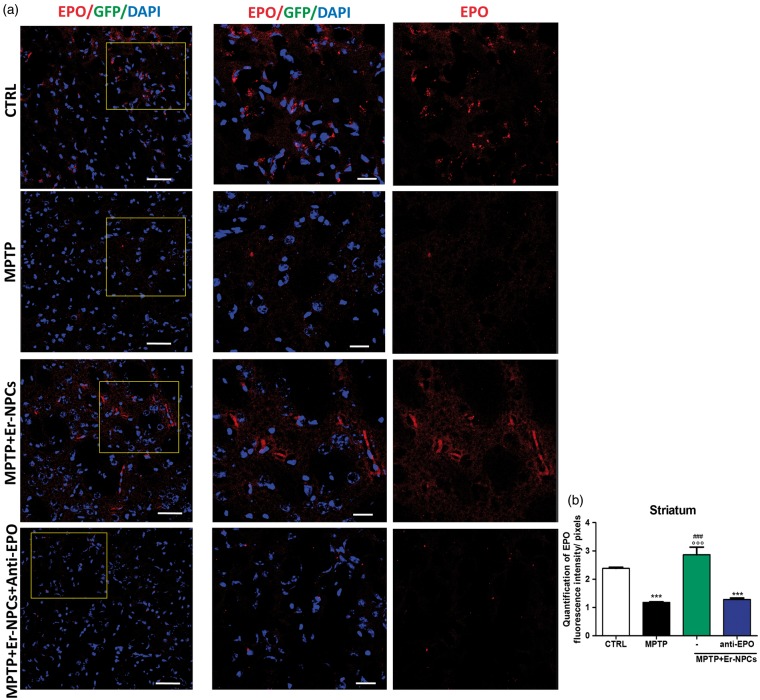
Er-NPCs injection preserves the EPO immunoreactivity in the striatum of MPTP animals. (a) Representative confocal images of striatal slices immunostained with EPO. Slices were obtained from brains of healthy mice (CTRL), mice injected with MPTP without cell treatment (MPTP) or treated with Er-NPCs injected alone (MPTP + Er-NPCs) or in presence of anti-EPO antibody (MPTP + Er-NPCs + anti-EPO). Scale bars represent 50 µm in the first lane and 20  µm in the second lane. (b) The graph reports the quantification of EPO fluorescence intensity made by ImageJ picture analysis software. For the groups MPTP + Er-NPCs and MPTP + Er-NPCs + anti-EPO, slices were chosen from striatal areas where injected Er-NPCs were absent. The analysis was performed by evaluating nine different fields for each condition (*n* = 3 mice each group; 3 fields or mouse). ***p < .001 versus CTRL, ###*p* < .001 versus anti-EPO, °°°*p* < .001 versus MPTP.

The Er-NPCs expression of EPO after the transplant was investigated by immunofluorescence in coronal section of striatum 2 weeks postinjection. Figure 7 shows that the great majority of transplanted Er-NPCs are positive to the staining for EPO. Although when Er-NPCs were coinjected with the anti-EPO antibody, the EPO expression resulted completely abrogated (Figure 7). Moreover, it is appreciable to note that Er-NPCs injected with anti-Epo antibody maintain an immature round morphology (Figure 7). However, EPO is expressed also in the host central nervous system where, according to our previous reports (Digicaylioglu et al., 1995; Carelli et al., 2016), it exerts neurotrophic and neuroprotective actions (Konishi et al., 1993; Digicaylioglu et al., 1995; Gorio et al., 2002; Marti, 2004; Jelkmann, 2011). The expression of EPO was then investigated by immunofluorescence in the striatum of MPTP animals, and in MPTP animals treated with Er-NPCs coinjected or not with anti-EPO antibody. To perform this analysis, striatum tissue sections were taken from areas neighboring the localization of the transplanted cells. The immune-reactivity was abundant in the striatum of healthy control animals (CTRL; Figure 8). The EPO expression resulted strongly abrogated in the degenerated striatum (MPTP; Figure 8), while its expression resulted completely preserved by the injection of Er-NPCs and, again, seemed strictly related to their physiologic capability to produce erythropietin (MPTP + Er-NPCs), as the coinjection of anti-EPO antibody strongly abolished the expression of EPO (MPTP + Er-NPCs + anti-EPO; Figure 8).

### Er-NPCs Transplantation Promotes Recovery of Function and Preserve TH Immunoreactivity in SN

**Figure 9.**
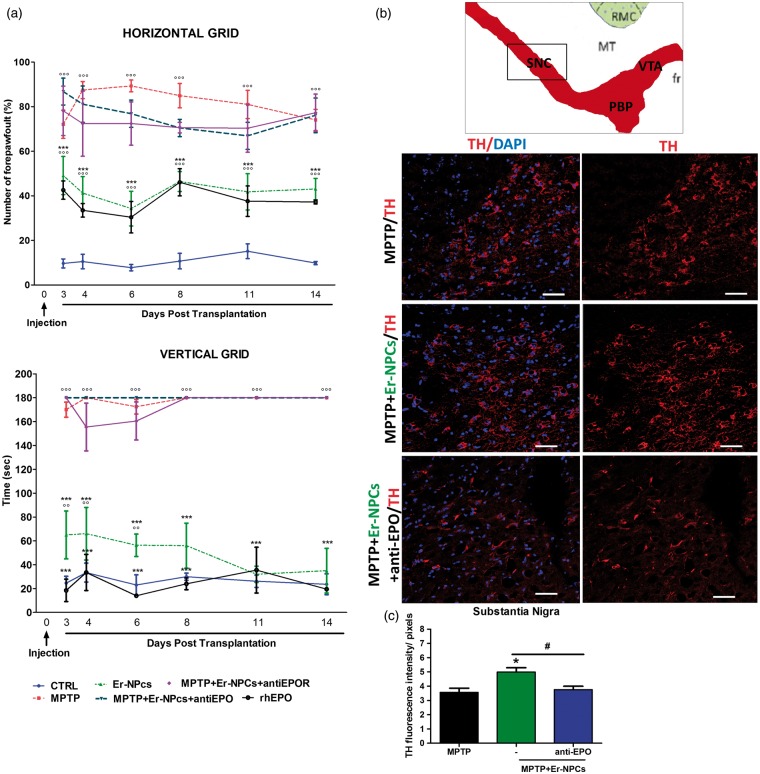
EPO released by transplanted Er-NPCs promotes their therapeutic effect. (a) Horizontal and vertical grid tests show the recovery of function in MPTP-intoxicated animals transplanted with Er-NPCs. Other groups of MPTP animals received specific antibodies against EPO (anti-EPO) and EPO receptor (anti-EPO R) in addition to Er-NPCs. rhEPO (1 U/g) was administered via the same route as positive control. Data are expressed as mean of three different experiments ± *SD* (*n* = 3 animals for each group in each experiment). Statistical differences were determined by means of one-way ANOVA test followed by Bonferroni posttest. °°°*p* < .001, °°*p* < .01 versus CTRL, ***p < .001 versus MPTP. (b) Representative TH staining of midbrain coronal sections of not-treated MPTP mice and MPTP mice grafted with Er-NPCs in presence or not of anti-EPO antibody. TH staining is showed in red and nuclei in blue (DAPI). Scale bars represent 50  µm. The graph reports the quantification of fluorescence intensity obtained by ImageJ picture analysis software. (c) The analysis was performed by evaluating nine different fields for each condition (*n* = 3 mice each group; 3 fields or mouse). *p < .05 versus CTRL and #*p* < .05 versus MPTP.

The therapeutic effect of intrastriatal injection of Er-NPCs was investigated also by evaluating the functional recovery by means of behavioral tests described earlier (see Figure 1(c) and Materials and Methods section). Three days after cell injection, the percentage of forepaw faults of Er-NPCs-injected mice was 34.26 ± 7.8% (horizontal grid test) compared with 89.3 ± 2.6% of not-treated MPTP animals (Figure 9(a)). The early improvement observed in Er-NPCs-transplanted mice was then maintained throughout the period of observation, while MPTP mice gradually worsened, reaching the maximum deficit at Day 8 (Figure 9(a)). The difference between Er-NPCs-treated and MPTP groups was even more conspicuous, when their motor coordination ability was evaluated by means of the vertical grid test. Three days after transplantation, Er-NPCs-injected animals showed a significant improvement in the time required for turning and descending the grid (62.7 ± 8.4 sec). While the time employed by MPTP mice was almost twice as long (112.7 ± 2.7 sec). The functional improvement promoted by Er-NPCs treatment steadily increased reaching values similar to controls (healthy mice) within 2 weeks after cells injection (Figure 9(a)). Er-NPCs inoculation was performed with coadministration of anti-EPO or anti-EPOR. Data show that the addition of either antibodies obliterated the positive effect of Er-NPCs administration described earlier (Figure 9(a)). Such a counteractive effect was observed with both behavioral motor tests. The administration of recombinant human-EPO (rhEPO; 1 U/g) in MPTP mice significantly improved the behavioral impairment as early as 3 days after injection in both tests, as Er-NPCs did (Figure 9(a)). The injection of anti-EPO and anti-EPOR antibodies alone did not modify the animal behavioral impairment caused by MPTP (Supplemental Table 1). These data suggest that the positive action of Er-NPCs in MPTP mice was likely due to EPO release by transplanted Er-NPCs. Moreover, the positive outcome due to Er-NPCs injection is stable for a long observational research period; so far, we monitored the functional recovery up to 50 days after Er-NPCs transplantation (Supplementary Figure 1). The histological analysis performed by immunofluorescence 2 weeks after transplantation shows that TH-positive cell bodies of the SN were very faintly detectable in MPTP mice, as expected (Figure 9(b) and (c)). The TH immunoreactivity was significantly higher after Er-NPCs transplantation (Figure 9(b) and (c)), and in the SN of anti-EPO coinjected mice, the TH immunoreactivity was comparable to MPTP group (Figure 9(b) and (c)). Tissue levels of DA and its metabolites were determined by high-performance liquid chromatography (see Materials and Methods section). MPTP exposure caused a 50% reduction of DA in both the left and right striatum that was maintained up to the end of experimentation period (24 days after first MPTP administration; Table 4). Such a loss was not modified by Er-NPCs transplantation (Table 4). Therefore, the behavioral recovery promoted by Er-NPCs occurs in absence of any restoration of DA, 3,4-dihydroxyphenylacetic acid, homovanillic acid, and 3-methoxytyramine that remained reduced and unaffected by the treatment with Er-NPCs (Table 4). Levels of NE, 5-HT, and 5-HIAA were also measured. In control healthy animals (CTRL), the striatal levels of these neurotransmitters were as follows: 8.74 ± 1.25 ng/mg proteins (NE), 24.02 ± 1.55 ng/mg proteins (5-HT), and 7.87 ± 0.37 (5-HIAA), respectively (Supplementary Table 2). Such levels were not affected neither by MPTP administration nor by the injection of Er-NPCs (Supplementary Table 2). Neurotransmitters were also quantitatively determined in frontal cortex and midbrain homogenates obtained from healthy (CTRL), MPTP, and MPTP + Er-NPCs animal groups, no significant differences were found among these groups (data not shown).

**Table 4.** Er-NPCs Injection Did Not Promote the Increase of DA and its Metabolites.

Right striatum	CTRL	MPTP	MPTP + Er-NPCs
DA	99.52 ± 11.32	48.28 ± 19.50 *p* < .001 versus CTRL	36.25 ± 11.07 *p* < .001 versus CTRL
DOPAC	8.57 ± 1.00	4.61 ± 2.36 *p* < .001 versus CTRL	3.90 ± 1.06 *p* < .001 versus CTRL
HVA	15.56 ± 2.02	7.68 ± 2.24 *p* < .001 versus CTRL	8.28 ± 1.84 *p* < .001 versus CTRL
3-MT	7.75 ± 1.18	6.71 ± 0.56	6.71 ± 2.10
Left striatum			
DA	99.52 ± 11.30	54.44 ± 4.65 *p* < .001 versus CTRL	32.32 ± 8.61 *p* < .001 versus CTRL
DOPAC	8.57 ± 1.00	6.09 ± 1.06 *p* < .001 versus CTRL	5.27 ± 1.13 *p* < .001 versus CTRL
HVA	15.56 ± 2.02	13.33 ± 3.03 *p* < .001 versus CTRL	10.03 ± 2.56 *p* < .001 versus CTRL
3-MT	7.75 ± 1.18	8.95 ± 1.62	6.15 ± 1.93

*Note.* DA = dopamine; DOPAC = 3,4-dihydroxyphenylacetic acid; HVA = homovanillic acid; 3-MT =3-methoxytyramine; CTRL = control; MPTP = 1-methyl-4-phenyl-1,2,3,6-tetrahydropyridine; Er-NPCs = erythropoietin-releasing neural precursors. The determination was performed at 15 days after cells transplantation by HPLC (see Materials and Methods section). Levels of DA, DOPAC, HVA, and 3-MT were determined separately in the left (ipsilateral to the injection) and right (contralateral to the injection site) striatum. Data are expressed in ng/mg of proteins as mean of two experiments ± *SD* (*n* = 5 mice in each group).

### Er-NPCs Transplantation Down-Regulate IL-6 mRNA Levels and Macrophages Invasion of the Striatum

**Figure 10.**
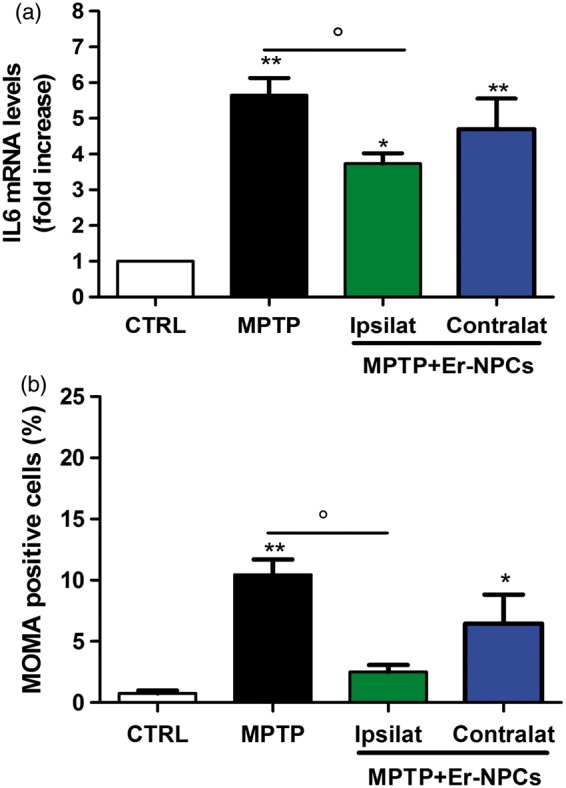
Er-NPCs transplantation down-regulates IL-6 mRNA levels and reduces macrophages invasion. (a) Expression of IL-6 mRNA levels was evaluated by real time RT-PCR. Each group was composed of three mice. Values represent the number of fold increase versus control and is reported as mean ± *SD*. *p < .05 versus CTRL, **p < .01 versus CTRL, and °*p* < .05 versus MPTP. (b) The graph reports the quantification of MOMA (mouse monocytes/macrophages marker) positive cells performed in striatal coronal slices of healthy (CTRL) mouse brain, MPTP mouse brain, and MPTP mouse brain injected with Er-NPCs (MPTP +Er-NPCs). The analysis was performed by evaluating three different fields for each condition. Date are reported as mean ± *SD*. **p < .01 versus CTRL, °°*p* < .01 versus MPTP, $$*p* < .01 versus contralateral.

Inflammation is part of the physiological response to MPTP as indicated by astroglia activation (Gao et al., 2003; Wirdefeldt et al., 2011). To investigate the Er-NPCs anti-inflammatory action, levels of IL-6 mRNA and monocytes/macrophages invasion were evaluated in both left and right striatum. The graph reported in panel A of Figure 10 shows a significant counteraction of IL-6 increase in the left striatum of Er-NPCs-treated MPTP mice (Figure 10(a)). Such effect was less marked in the right striatum (contralateral to the transplant). In addition, the striatal invasion by macrophages, evaluated by staining with anti-MOMA (monocytes/macrophages) in both left and right striatum, was counteracted by Er-NPCs transplantation (Figure 10(b)).

## Discussion

This study was aimed at evaluating the fate and effects of transplanted EPO-releasing neural precursors (Er-NPCs) in the MPTP experimental mouse model of PD. Here, we report that, 2 weeks after transplantation, Er-NPCs are differentiating with expression of neural markers and migrate from the injection site throughout the striatum by following dorso-ventral and rostro-caudal pathways to reach ipsilateral and contralateral SNpc. In addition, such a single administration of Er-NPCs improves the motor deficits evaluated by horizontal and vertical grid tests. All of the described features depend on autocrine EPO production and release by Er-NPCs, as their co-injection with anti-EPO or anti-EPO receptor antibodies abrogates these favorable effects.

Er-NPCs are adult neural stem cells isolated from subventricular zone 6 hr after death of the mouse donor (Marfia et al., 2011). These cells, when infused in tail vein, after experimental traumatic spinal cord injury migrate to lesion site, attenuate secondary degeneration, and significantly ameliorate recovery of function (Carelli et al., 2014, 2015). In addition, Er-NPCs survive and could be found even long time (90 days) after transplantation in such a strongly unfavorable environment; where they differentiate into cholinergic neuronal-like cells and counteract posttraumatic neuroinflammation (Carelli et al., 2014, 2015).

So far, fetal tissue transplantation procedures are the most studied stem cells transplantation approach to cure PD (Tsui and Isacson, 2001; Barker et al., 2013; Barker, 2014). Despite the wake of a long series of encouraging open-label studies, the initial enthusiasm for such procedures almost vanished when two double-blind placebo-controlled clinical trials showed only moderate symptomatic improvements that were accompanied by severe disabling dyskinesias (Tsui and Isacson, 2001; Barker, 2014). To overcome such negative outcomes, recent work has been centered on the generation of TH-positive neurons from ESCs (Ortiz et al., 2007; Cai et al., 2009), embryonic neural stem cells, mesenchymal stroma cells, and more recently pluripotent stem cells, in order to be applied in experimental models of PD (Kim et al., 2002; Barzilay et al., 2009; Friling et al., 2009; Hallett et al., 2015; Pollock et al., 2016).

Here, we describe that, after intrastriatal injection, more than 75% of Er-NPCs survive, integrate into the striatum, reach a mature morphological phenotype, and differentiate mostly in neuronal-like cells. Such differentiation is higher for those cells that migrate to more distal sites from the injection point: 60% MAP-2 positive and above than 80% NeuN-positive. Most of Er-NPCs differentiated in TH positive cells, and a large percentage of these were positive for ChAT and GABA. Er-NPCs remaining in the area of injection show a round morphology accompanied by the nestin expression. Differently from what had been previously observed, with the transplantation of embryonic MGE precursors cells into adult rat striatum (Martinez-Cerdeno et al., 2010), the migratory pathways of Er-NPCs seem to be defined by following a linear route, as these cells did not spread in other parts within the striatum. Moreover, Er-NPCs had migrated across the striatum and reached the SN where they integrate. It therefore appears that some specific signals capable of directing the cellular migratory process must exist and this seemed in strict relation to the release of EPO by Er-NPCs. The EPO neuroprotective and anti-inflammatory effects are very well known and supported by a large body of literature, showing that *in vivo* administration of EPO promotes recovery of function after central nervous system lesions (Gorio et al., 2002; Brines and Cerami, 2008). Also in rat models of PD, it has been shown that the administration of rhEPO or EPO analogs had neuroprotective and neuro-rescue effects (Thomas et al., 2013; Jia et al., 2014; Erbaş et al., 2015). Therefore, it is conceivable that the release of such a neuroprotective factor by Er-NPCs may also have a favorable impact on the striatum dopaminergic functions, and consequently, on behavioral recovery. Ten days after MPTP administration, before the injection of Er-NPCs, mice showed important behavioral deficits that were quickly reverted by Er-NPCs transplantation. At Day 8 after Er-NPCs injection, the behavioral parameters were comparable to that of healthy control animals. Such a recovery was independent from any improvement in striatal DA content, since the loss, caused by MPTP, remained unaltered after transplantation of Er-NPCs. It is therefore clear that the protective or reparative action of Er-NPCs is not mediated by the recovery of DA levels in the striatum, but likely by an improved efficacy of the remaining DA in the striatum under the trophic action of Er-NPCs-released factors such as EPO. These may lead to an enhanced efficacy of the synaptic dopaminergic transmission by an action exerted on striatal and SN microenvironment favoring the survival of dopaminergic cell bodies and striatal axon terminals. It is conceivable that the suppression of the MPTP-mediated inflammation may have increased the efficacy of the remaining DA (Hantraye et al., 1992). Indeed, a preservation of TH labeling can be observed in both brain areas, and this is accompanied by a strong preservation of EPO expression in the striatum of grafted animals. It should also be noted that Er-NPCs migrate to SN ipsilateral and contralateral to the injection site, and this may have affected SN neuroinflammation.

Since Er-NPCs-mediated behavioral recovery was prevented by the intrastriatal blockade of EPO or EPOR, and that the direct EPO-administration into MPTP affected striatum promoted a prompt behavioral recovery, we believe that EPO may be one of the effector of Er-NPCs transplants. This may modify the striatal microenvironment thanks to a dual effect. First, due to the modulation of the migratory response by endogenous stem cells toward the area of Er-NPCs accumulation. EPO has been shown to direct the migration of endogenous stem cells toward EPO-reach source (Shingo et al., 2000; Erbaş et al., 2015). Second, the mechanism could be related to a local counteraction of inflammation and neuroprotective action (Agnello et al., 2002; Gorio et al., 2002; Chen et al., 2007) on neurons and neural processes affected by MPTP. The results of such actions may be the reexpression of TH by MPTP-lesioned SN neurons. A similar effect was observed after a septo-hippocampal lesion, where septal neurons recovered ChAT positivity following NGF or BDNF infusion (Morse et al., 1993). Our previous work in experimental traumatic spinal cord injury reported the reduction of inflammatory cytokines levels, reactive GFAP-positive gliosis, and macrophage infiltration after the treatment with rhEPO (Gorio et al., 2002) or administration of Er-NPCs (Carelli et al., 2014, 2015). This seems to happen also in the present work by the counteraction of inflammation events caused by MPTP, particularly ipsilateral to the injection site. These counteractive effects on astrocytes are quite relevant, since these cells are positively activated in PD and produce an elevated level of inflammatory cytokines and reactive oxygen species (Block and Hong, 2007). Moreover, such an activation is correlated with increased neural death (McGeer et al., 1988; Sofroniew, 2005). Future studies will be needed to understand in deep the physiological integration of Er-NPCs after transplantation into the adult striatum. Our previous work performed *in vitro* showed that Er-NPCs are able to differentiate in GFAP-positive cells (Marfia et al., 2011). However, *in vivo*, after transplantation in experimental model of traumatic spinal cord injury, Er-NPCs differentiated mostly in neurons and did not show GFAP positivity (Carelli et al., 2015). For the future, further experiments need to be done to investigate whether transplanted Er-NPCs in PD experimental models could differentiate into astrocytes or play any role in striatal astrogliosis.

## Conclusions

Er-NPCs markedly improve morphological, biochemical, and behavioral dysfunctions caused by MPTP intoxication. This effect is likely mediated by local EPO release, which is also effective when applied directly, and the mitigation of the neuro-inflammatory events may be the key factor for achieving recovery of function. Therefore, transplantable cells capable of releasing anti-inflammatory, neuroprotective, and neurothrophic factors may represent a promising path for the treatment of neurodegenerative disorders (Pollock et al., 2016), and in this specific case, EPO may be effective.

## Summary

Neural precursors naturally releasing EPO were applied to an MPTP-induced mouse model of Parkinson’s disease. These cells differentiated and migrated in the brain areas affected by the disease and promoted recovery of function.

## Supplementary Material

Supplementary material

## Supplementary Material

Supplementary material

## Supplementary Material

Supplementary material
