# Supplementary material for: Grafted Neural Precursors Integrate Into Mouse Striatum, Differentiate and Promote Recovery of Function Through Release of Erythropoietin in MPTP-Treated Mice
Source: ASN Neuro. 2016 Oct 27;8(5):1759091416676147. doi: 10.1177/1759091416676147 (PMC5102092; doi:10.1177/1759091416676147)
Supplement: Supplementary material [file Supplementary_Figure_1.pdf]

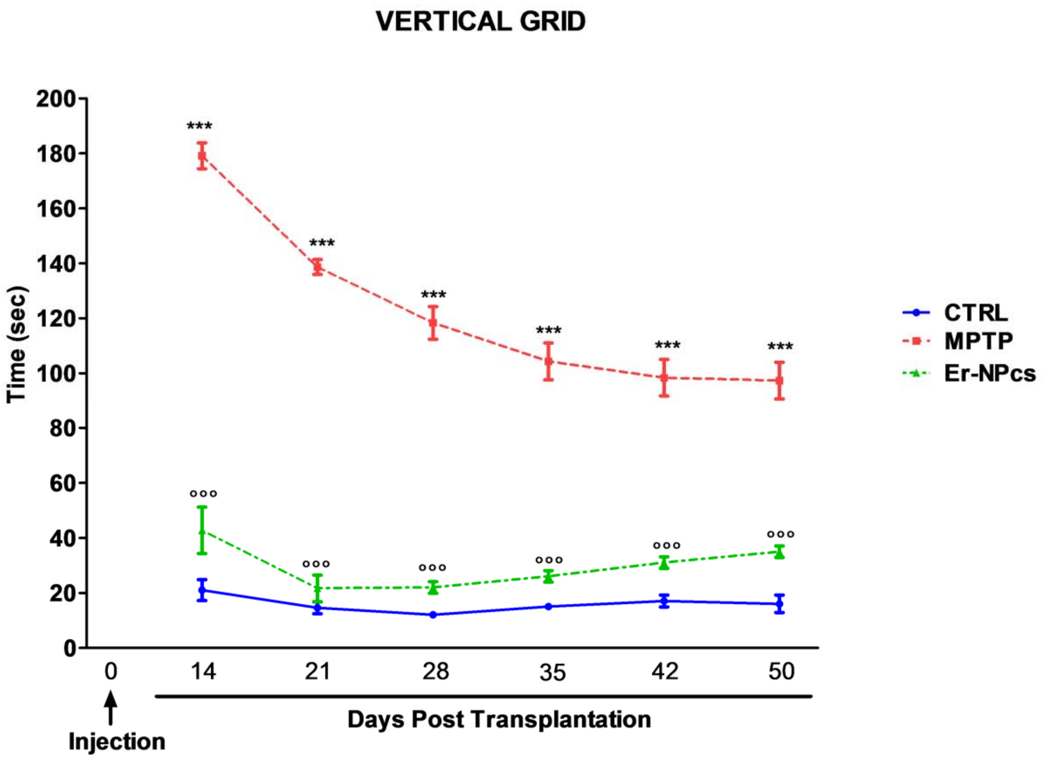

Supplementary Figure 1

Er-NPCs action is stable for a long observational research period. Vertical grid test showing the stability of recovery in MPTP animals transplanted with Er-NPCs. Data are expressed as mean of two different experiments ± SD (n=3 animals for each group in each experiment). Statistical differences were determined by means of one-way ANOVA test followed by Bonferroni post-test. °°°$p < 0.001$ vs MPTP; ***$p < 0.001$ vs CTRL.
