# Supplementary material for: Grafted Neural Precursors Integrate Into Mouse Striatum, Differentiate and Promote Recovery of Function Through Release of Erythropoietin in MPTP-Treated Mice
Source: ASN Neuro. 2016 Oct 27;8(5):1759091416676147. doi: 10.1177/1759091416676147 (PMC5102092; doi:10.1177/1759091416676147)
Supplement: Supplementary material [file Supplementary_Table_1.pdf]

Supplementary Table 1

Row Data relative to behavioral evaluation performed in specific animal groups. Horizontal and vertical grid tests performed in MPTP + anti-EPO, MPTP + anti-EPOR, MPTP + albumin. Data are expressed as mean of three different experiments ± SD (n=3 animals for each group in each experiment). Statistical differences were determined by means of one-way ANOVA test followed by Bonferroni post-test.

Horizontal grid

Days post transplant	MPTP+anti-EPO	MPTP+anti-EPOR	MPTP+Albumin
**3**	75.16 ± 5.81 (p<0.001 vs CTRL, MPTP+Er-NPCs)	76.36 ± 8.87 (p<0.001 vs CTRL, MPTP+Er-NPCs)	85 ± 0 (p<0.001 vs CTRL, MPTP+Er-NPCs)
**4**	87.08 ± 5.37 (p<0.001 vs CTRL, MPTP+Er-NPCs)	97.60 ± 7.95 (p<0.001 vs CTRL, MPTP+Er-NPCs)	100 ± 5.6 (p<0.001 vs CTRL, MPTP+Er-NPCs)
**6**	77.98 ± 5.53 (p<0.001 vs CTRL, MPTP+Er-NPCs)	93.80 ± 2.24 (p<0.001 vs CTRL, MPTP+Er-NPCs)	100 ± 0 (p<0.001 vs CTRL, MPTP+Er-NPCs)
**8**	70.59 ± 2.38 (p<0.001 vs CTRL, MPTP+Er-NPCs)	81.01 ± 3.19 (p<0.001 vs CTRL, MPTP+Er-NPCs)	87.43 ± 1.02 (p<0.001 vs CTRL, MPTP+Er-NPCs)
**11**	87.15 ± 9.17 (p<0.001 vs CTRL, MPTP+Er-NPCs)	80.44 ± 5.63 (p<0.001 vs CTRL, MPTP+Er-NPCs)	87.5 ± 1.32 (p<0.001 vs CTRL, MPTP+Er-NPCs)
**14**	91.67 ± 5.00 (p<0.001 vs CTRL, MPTP+Er-NPCs)	91.35 ± 5.00 (p<0.001 vs CTRL, MPTP+Er-NPCs)	80 ± 0.98 (p<0.001 vs CTRL, MPTP+Er-NPCs)

Vertical grid.

Days post transplant	MPTP+anti-EPO	MPTP+anti-EPOR	MPTP+Albumin
**3**	176.66 ± 3.33 (p<0.001 vs CTRL, MPTP+Er-NPCs)	180 ± 5.31 (p<0.001 vs CTRL, MPTP+Er-NPCs)	180 ± 6.28 (p<0.001 vs CTRL, MPTP+Er-NPCs)
**4**	160.75 ± 22.23 (p<0.001 vs CTRL, MPTP+Er-NPCs)	175 ± 5.77 (p<0.001 vs CTRL, MPTP+Er-NPCs)	180 ± 7.45 (p<0.001 vs CTRL, MPTP+Er-NPCs)
**6**	180 ± 8.10 (p<0.001 vs CTRL, MPTP+Er-NPCs)	180 ± 9.67 (p<0.001 vs CTRL, MPTP+Er-NPCs)	180 ± 10.12 (p<0.001 vs CTRL, MPTP+Er-NPCs)
**8**	180 ± 4.33 (p<0.001 vs CTRL, MPTP+Er-NPCs)	180 ± 6.55 (p<0.001 vs CTRL, MPTP+Er-NPCs)	180 ± 8.99 (p<0.001 vs CTRL, MPTP+Er-NPCs)
**11**	180 ± 8.14 (p<0.001 vs CTRL, MPTP+Er-NPCs)	180 ± 7.91 (p<0.001 vs CTRL, MPTP+Er-NPCs)	180 ± 6.88 (p<0.001 vs CTRL, MPTP+Er-NPCs)
**14**	180 ± 7.52 (p<0.001 vs CTRL, MPTP+Er-NPCs)	180 ± 6.28 (p<0.001 vs CTRL, MPTP+Er-NPCs)	180 ± 9.16 (p<0.001 vs CTRL, MPTP+Er-NPCs)
