# Supplementary material for: Grafted Neural Precursors Integrate Into Mouse Striatum, Differentiate and Promote Recovery of Function Through Release of Erythropoietin in MPTP-Treated Mice
Source: ASN Neuro. 2016 Oct 27;8(5):1759091416676147. doi: 10.1177/1759091416676147 (PMC5102092; doi:10.1177/1759091416676147)
Supplement: Supplementary material [file Supplementary_Table_2.pdf]

Supplementary Table 2

NE, 5-HT and 5-HIAA quantification. The determination was performed at 15 days after cells transplantation by HPLC (see M&M). Levels of neurotransmitters were determined separately in the left (ipsilateral to the injection) and right (contralateral to the injection site) striatum. Data are expressed in ng/mg of proteins as mean of two experiments ± SD (n = 5 mice in each group).

**Right striatum**	**CTRL**	**MPTP**	**MPTP+Er-NPCs**
**NE**	8.74 ± 1.25	6.34 ± 2.66	8.80 ± 3.83
**5-HT**	24.02 ± 1.55	20.88 ± 1.27	23.56 ± 4.97
**5-HIAA**	7.87 ± 0.37	7.44 ± 1.10	8.09 ± 1.59

**Left striatum**	**CTRL**	**MPTP**	**MPTP + Er-NPCs**
**NE**	8.74 ± 1.25	14.36 ± 7.36	7.26 ± 4.48
**5-HT**	24.02 ± 1.54	26.90 ± 6.61	19.89 ± 5.52
**5-HIAA**	7.87 ± 0,37	7.35 ± 0.72	7.22 ± 2.02
